# Uplink NOMA-MIMO Systems with Rayleigh Power Distribution

**DOI:** 10.3390/s22114002

**Published:** 2022-05-25

**Authors:** Mikhail Bakulin, Taoufik Ben Rejeb, Vitaly Kreyndelin, Denis Pankratov, Aleksei Smirnov

**Affiliations:** Moscow Technical University of Communications and Informatics (MTUCI), Moscow 111024, Russia; m.g.bakulin@gmail.com (M.B.); vitkrend@gmail.com (V.K.); dpankr@mail.ru (D.P.); smirnov.al.ed@gmail.com (A.S.)

**Keywords:** MIMO, PD-NOMA, load factor, channel capacity

## Abstract

The article is devoted to multiple-input multiple-output antenna systems, also called MIMO systems, which are widely used in wireless communication systems. In this article we consider a case when the MIMO system works in overloaded mode. In this mode MIMO systems can be considered as a system with non-orthogonal multiple access NOMA. The main goal of this article is to analyze this interesting case using statistical computer simulation. Based on the analysis of the capacity of a discrete-continuous multiuser MIMO uplink channel, the possibility of such systems functioning in overload mode is proved.

## 1. Introduction

Non-Orthogonal Multiple Access (NOMA) is a key technology for future wireless systems including 6G mobile networks [1,2,3,4,5,6]. Compared with orthogonal multiple access technologies, NOMA have a new parameter of communication system–load factor.

The load factor is a ratio between the number of subscribers (or the total number of data streams distributed using resources) with the number of orthogonal resources used. For systems with orthogonal access, it does not exceed 1 (<100%). For NOMA systems in full load mode, it must be greater than 1 (>100%). Therefore, for NOMA systems, this indicator is more often referred to as the overload coefficient of the communication system.

MIMO (Multiple-Input Multiple-Output) is another known and efficient technology widely used in modern mobile communication systems [4,5,6,7,8,9,10,11]. MIMO technology improves energy and spectral efficiency and allows an increase in the capacity and bandwidth of wireless systems and the data rate of each user.

The load factor also can be used for the analysis single-user mode MIMO (Single User MIMO-SU-MIMO) and for multiuser mode MIMO (Multiuser MIMO-MU-MIMO) systems as well as for NOMA systems [12]. It is shown in [8] that MIMO channel can be represented as a combination of orthogonal channels, i.e., orthogonal resources, with different characteristics.

The number of such orthogonal resources is determined by the minimum number $\min\left(N_{tx,\Sigma },\;N_{rx,\Sigma }\right)$, where $N_{tx,\Sigma },\;N_{rx,\Sigma }$ is the total number of transmitting and receiving antennas in the MIMO system, respectively. In the single-user mode, overloading means that the number of streams distributed over the transmitting antennas is greater than the number of receiving antennas. In the multiuser mode, overloading occurs when the total number of distributed streams across all transmitting antennas is greater than the number of receiving antennas $N_{tx,\Sigma }$ at the base station.

Theoretical analysis of the MIMO channel has shown that the best results are obtained when the number of receiving antennas at the base station $N_{rx}$ is greater or equal to the total number of multiplexed data streams of all users, which is usually equal to the total number of transmitting antennas of all users $N_{tx,\Sigma }$ [6,11,12,13,14,15,16]. This conclusion has been originally obtained for a single user system [8], where it is shown that when the condition $N_{rx}\geq N_{tx}$ is met, the system bandwidth increases linearly from the number of transmitting antennas. Therefore, we have a conclusion that MIMO systems, where $N_{tx,\Sigma }>N_{rx}$, are ineffective.

Let us consider a case when $N_{tx,\Sigma }=N_{rx}$ corresponds to 100% of the MIMO system load. Then the case $N_{tx,\Sigma }>N_{rx}$ can be considered as the operation of the system in overload mode. In this case, the MU-MIMO system becomes a NOMA system. Moreover, this case can be considered as a NOMA system with power division (Power Domain NOMA-PD-NOMA) [6]. In this article it will be shown that MU-MIMO systems with independent Rayleigh fading in an uplink channel can work quite efficiently in overload mode because in this case NOMA technology is implemented with a natural separation of signals in the power domain.

## 2. System Model

Let us consider a multiuser system with a MIMO channel, in which every *u*-th user has $N_{tx,u}$ transmitting antennas. The base station has $N_{rx}$ receiving antennas. Each user uses spatial multiplexing with the maximum use of the number of transmitting antennas, i.e., the number of multiplexed streams of each user is equal to the number of transmitting antennas. In this case, the system model is described by the following equation:

(1) $$Y=\sum_{u=1}^{U}\frac{\sqrt{P_{u}}}{\sqrt{N_{tx,u}}}H_{u}X_{u}\left(\Theta_{u}\right)+\eta$$

where U-number of users; $P_{u},N_{tx,u}$-power and number of transmitting antennas of *u*-th user; Y-complex vector of received signal dimension of $N_{rx}$; $H_{u}$-matrix of *u*-th user MIMO channel dimension of $\left(N_{rx}\times N_{tx,u}\right)$; $X_{u}\left(\Theta_{u}\right)={\left[\begin{array}{cccc} x_{1,u}\left(\theta_{1,u}\right) & x_{2,u}\left(\theta_{2,u}\right) & \ldots & x_{N_{tx,u},u}\left(\theta_{N_{tx,u},u}\right) \end{array}\right]}^{T}$-vector of QAM symbols of *u*-th user dimension of $N_{tx,u}$, where each element is a function $x_{i,u}\left(\theta_{i,u}\right)$ of discrete parameter $\theta_{i,u}\in \Omega_{i,u}$, $\Omega_{i,u}$-set of values of *i*-th discrete parameter of *u*-th user, $i=\bar{1,N_{tx,u}},\;\;\;u=\bar{1,U}$; $\Theta_{u}={\left[\begin{array}{ccc} \theta_{1,u} & \ldots & \theta_{N_{tx,u},u} \end{array}\right]}^{T}$-vector of random discrete values dimension of $N_{tx,u}$; $\eta ∼\mathbb{C}\mathbb{N}\left(0,2\sigma_{\eta }^{2}I_{N_{rx}}\right)$-complex vector of additive white Gaussian noise in channel dimension of $N_{rx}$. Functional dependencies $x_{i,u}\left(\cdot \right)$ describing the constellations of used QAM symbols can be either the same for all i and u or different. The discrete parameter $\theta_{i,u}$ is a function of binary symbols vector (information bits). The dimension of the set $\Omega_{i,u}$ is determined by the number of bits $k_{b,i,u}$ transmitted by the *i*-th symbol of *u*-th user and is equal to $2^{k_{b,i,u}}$.

For future simplification, we assume that the number of transmitting antennas for each user is the same $N_{tx,u}=N_{tx}$, in addition, all users use the same power of the emitted signals and the same constellations, i.e., $P_{u}=P$, $x_{i,u}\left(\theta_{i,u}\right)=x\left(\theta_{i,u}\right)$, $k_{b,i,u}=k_{b}$, $\Omega_{i,u}=\Omega$.

Using this simplification, Equation (1) can be rewritten as follows:

(2) $$Y=\frac{\sqrt{P}}{\sqrt{N_{tx}}}HX\left(\Theta \right)+\eta$$

where $H≜\left[\begin{array}{ccc} H_{1} & \ldots & H_{U} \end{array}\right]$ -extended matrix of uplink multiuser MIMO channel, $X\left(\Theta \right)≜{\left[\begin{array}{ccc} x\left(\theta_{1,1}\right) & \ldots & x\left(\theta_{N_{tx},1}\right) \end{array}\begin{array}{ccc} x\left(\theta_{1,2}\right) & \ldots & x\left(\theta_{N_{tx},2}\right) \end{array}\ldots x\left(\theta_{1,U}\right)\ldots x\left(\theta_{N_{tx},U}\right)\right]}^{T}$-extended vector of QAM symbols of all users dimension of $N_{tx,\Sigma }=UN_{tx}$. The number of possible vector states $X\left(\Theta \right)\in \Omega^{N_{tx,\Sigma }}$ is equal to the number of states of the total vector of discrete values, which is equal to $M=2^{N_{tx,\Sigma }k_{b}}=2^{N_{tx}k_{b}U}$.

Capacity of MIMO channel obtained in [7,8] is determined by the mutual information of the continuous channel:

(3) $$C=\underset{p\left(X\right)}{\max}I\left(X;Y\right)$$

where p(X)-a priori distribution of continuous vector X; I(X;Y)-mutual information between the input vector X and output vector of MIMO-channel Y, which is defined as:

(4) $$I(X,Y)=\int_{Y}\int_{X}p\left(Y|X\right)p\left(X\right)\log\left(\frac{p\left(Y|X\right)}{p\left(Y\right)}\right)dYdX$$

Maximization in Equation (3) is implemented over all possible a priori distributions. As is known, the maximum of this expression is observed with a Gaussian a priori distribution. As a result, for model (2), the capacity of continuous channel will be determined by the following equation:

(5) $$C=\log\det\left(I_{N_{rx}}+\frac{P}{2\sigma_{\eta }^{2}N_{tx}}HH^{\prime }\right)$$

Capacity of MIMO channel is a function of the channel matrix, i.e., C(H) which consists of random elements that depend on the type of fading and their correlation. Therefore, to analyze the average capacity (ergodic capacity) of the system with the MIMO channel, an averaging procedure is used for the distribution of random channel multipliers:

(6) $$C_{E}=E_{H}\left\{\log\det\left(I_{N_{rx}}+\frac{P}{2\sigma_{\eta }^{2}N_{tx}}HH^{\prime }\right)\right\}$$

Expression (6) can be written in another form, known as the multiple access capacity [14,15,17]:

(7) $$C_{E}=E_{H}\left\{\log\det\left(I_{N_{rx}}+\frac{P}{2\sigma_{\eta }^{2}N_{tx}}\sum_{u=1}^{U}H_{u}H_{u}{}^{\prime }\right)\right\}$$

**Figure 1:**
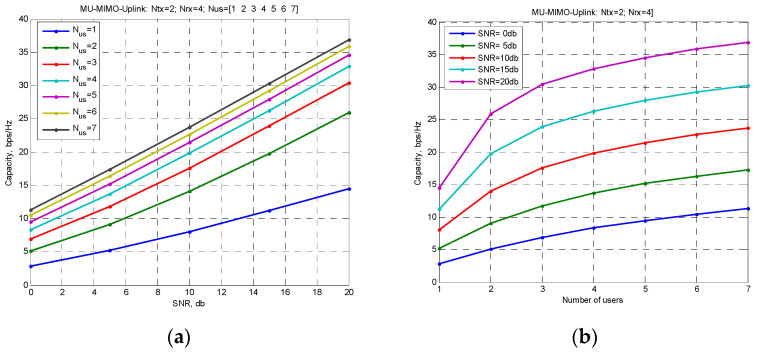
Dependencies of the MU-MIMO uplink channel capacity for continuous channel: (**a**) capacity dependencies on the SNR ratio for a different number of users, (**b**) capacity dependencies on the number of users for different values of the SNR ratio.

Figure 1 shows the capacity dependencies on the SNR ratio for a different number of users (Figure 1a) and on the number of users for different values of the SNR ratio (Figure 1b). Figures are obtained for values $N_{rx}=4$ and $N_{tx}=2$.

Figure 1 shows if $N_{tx,\Sigma }=UN_{tx}>N_{rx}$ the growth of capacity with an increase of number of users slows down and at high SNR ratios it becomes proportional to logU. These results are known, and from them, as a rule, it follows that the use of systems with an MIMO channel for the case is inefficient and is not advisable.

This conclusion is valid if we limit ourselves to considering the maximum capacity of a continuous channel as a criterion. In real systems, for information transmission, we use a discrete alphabet, i.e., the real channel is discrete–continuous. Therefore, it is interesting to analyze the capacity of the discrete–continuous channel.

## 3. Capacity of Discrete–Continuous MU-MIMO Uplink Channel

For a discrete–continuous channel, mutual information is described by the following Equation [9]:

(8) $$I\left(X;Y\right)=\sum_{i=1}^{M}\int_{Y}p\left(Y|X_{i}\right)p\left(X_{i}\right)\log\left(\frac{p\left(Y|X_{i}\right)}{p\left(Y\right)}\right)dY$$

Mutual information as for a continuous channel can be represented as an entropy difference:

(9) I(X;Y)=H(Y)−H(Y|X)

where:

(10) $$\begin{array}{l} H\left(Y\right)=-\int_{Y}p\left(Y\right)\log\left(p\left(Y\right)\right)dY\\ H\left(Y|X\right)=\sum_{i=1}^{M}p\left(X_{i}\right)H\left(Y|X_{i}\right)\\ H\left(Y|X_{i}\right)=-\int_{Y}p\left(Y|X_{i}\right)\log\left(p\left(Y|X_{i}\right)\right)dY\\ p\left(Y\right)=\sum_{i=1}^{M}p\left(Y|X_{i}\right)p\left(X_{i}\right) \end{array}$$

The bandwidth of a discrete–continuous channel is the maximum of mutual information according to an a priori discrete distribution:

(11) $$C=\underset{p\left(X_{i}\right),\bar{i=1,M}}{\max}I\left(X;Y\right)$$

It is known that the maximum entropy has a source of discrete information with a uniform a priori distribution, i.e., $p\left(X_{i}\right)=\frac{1}{M}$. In this case we have:

(12) $$\begin{array}{l} H\left(Y\right)=-\frac{1}{M}\sum_{i=1}^{M}\int_{Y}p\left(Y|X_{i}\right)\log\left(\frac{1}{M}\sum_{j=1}^{M}p\left(Y|X_{j}\right)\right)dY\\ H\left(Y|X_{i}\right)=\frac{1}{M}\sum_{i=1}^{M}H\left(Y|X_{i}\right) \end{array}$$

From model (2) we have:

(13) $$p\left(Y|X_{i}\right)=\frac{1}{{\left(2\pi \right)}^{N_{rx}}\sigma_{\eta }^{2N_{rx}}}\exp\left(-\frac{1}{2\sigma_{\eta }^{2}}{\left(Y-\tilde{H}X_{i}\right)}^{\prime }\left(Y-\tilde{H}X_{i}\right)\right)$$

where $X_{i}≜X\left(\Theta_{i}\right)$-*i*-th value of extended QAM symbols vector of all users, $i=\bar{1,M}$; $\tilde{H}≜\frac{\sqrt{P}}{\sqrt{N_{tx}}}H$.

Taking into account Equation (13), we have:

(14) $$\begin{array}{l} H\left(Y|X_{i}\right)=N_{rx}+N_{rx}\log\left(2\pi \sigma_{\eta }^{2}\right)\\ H\left(Y\right)=N_{rx}\log\left(\left(2\pi \sigma_{\eta }^{2}\right)\right)-\\ \frac{1}{M}\sum_{i=1}^{M}\int_{Y}\frac{1}{{\left(2\pi \sigma_{\eta }^{2}\right)}^{N_{rx}}}\exp\left(-\frac{{\left\|Y-\tilde{H}X_{i}\right\|}^{2}}{2\sigma_{\eta }^{2}}\right)\log\left(\frac{1}{M}\sum_{j=1}^{M}\exp\left(-\frac{{\left\|Y-\tilde{H}X_{j}\right\|}^{2}}{2\sigma_{\eta }^{2}}\right)\right)dY \end{array}$$

As a result, the bandwidth of a discrete-continuous MIMO channel will be determined by the following equation:

(15) $$\begin{array}{l} C=-\frac{1}{M}\sum_{i=1}^{M}\int_{Y}\frac{1}{{\left(2\pi \sigma_{\eta }^{2}\right)}^{N_{rx}}}\exp\left(-\frac{{\left\|Y-\tilde{H}X_{i}\right\|}^{2}}{2\sigma_{\eta }^{2}}\right)\log\left(\frac{1}{M}\sum_{j=1}^{M}\exp\left(-\frac{{\left\|Y-\tilde{H}X_{j}\right\|}^{2}}{2\sigma_{\eta }^{2}}\right)\right)dY-N_{rx}=\\ \log M-N_{rx}-\frac{1}{M}\sum_{i=1}^{M}\int_{Y}\frac{1}{{\left(2\pi \sigma_{\eta }^{2}\right)}^{N_{rx}}}\exp\left(-\frac{{\left\|Y-\tilde{H}X_{i}\right\|}^{2}}{2\sigma_{\eta }^{2}}\right)\log\left(\sum_{j=1}^{M}\exp\left(-\frac{{\left\|Y-\tilde{H}X_{j}\right\|}^{2}}{2\sigma_{\eta }^{2}}\right)\right)dY \end{array}$$

Transform the following expression:

$$\begin{array}{l} -\frac{1}{M}\sum_{i=1}^{M}\int_{Y}\frac{1}{{\left(2\pi \sigma_{\eta }^{2}\right)}^{N_{rx}}}\exp\left(-\frac{{\left\|Y-\tilde{H}X_{i}\right\|}^{2}}{2\sigma_{\eta }^{2}}\right)\log\left(\sum_{j=1}^{M}\exp\left(-\frac{{\left\|Y-\tilde{H}X_{j}\right\|}^{2}}{2\sigma_{\eta }^{2}}\right)\right)dY=\\ -\frac{1}{M}\sum_{i=1}^{M}\int_{Z_{i}}\frac{1}{{\left(2\pi \sigma_{\eta }^{2}\right)}^{N_{rx}}}\exp\left(-\frac{{\left\|Z_{i}\right\|}^{2}}{2\sigma_{\eta }^{2}}\right)\log\left(\sum_{j=1}^{M}\exp\left(-\frac{{\left\|Z_{i}-\tilde{H}\Delta X_{ji}\right\|}^{2}}{2\sigma_{\eta }^{2}}\right)\right)dZ_{i} \end{array}$$

where $Z_{i}≜Y-\tilde{H}X_{i}$.

Taking into account the transformations made, as a result we have done:

(16) $$C=\log M-N_{rx}-\frac{1}{M}\sum_{i=1}^{M}\int_{Z_{i}}\frac{1}{{\left(2\pi \sigma_{\eta }^{2}\right)}^{N_{rx}}}\exp\left(-\frac{{\left\|Z_{i}\right\|}^{2}}{2\sigma_{\eta }^{2}}\right)\log\left(\sum_{j=1}^{M}\exp\left(-\frac{{\left\|Z_{i}-\tilde{H}\Delta X_{ji}\right\|}^{2}}{2\sigma_{\eta }^{2}}\right)\right)dZ_{i}$$

At high SNR ratios the following approximation can be used:

(17) $$\begin{array}{l} \exp\left(-\frac{{\left\|Z_{i}\right\|}^{2}}{2\sigma_{\eta }^{2}}\right)\log\left(\sum_{j=1}^{M}\exp\left(-\frac{{\left\|Z_{i}-\tilde{H}\Delta X_{ji}\right\|}^{2}}{2\sigma_{\eta }^{2}}\right)\right)=\\ \exp\left(-\frac{{\left\|Z_{i}\right\|}^{2}}{2\sigma_{\eta }^{2}}\right)\log\left(\exp\left(-\frac{{\left\|Z_{i}\right\|}^{2}}{2\sigma_{\eta }^{2}}\right)+\sum_{\begin{array}{l} j=1\\ i\neq j \end{array}}^{M}\exp\left(-\frac{{\left\|Z_{i}-\tilde{H}\Delta X_{ji}\right\|}^{2}}{2\sigma_{\eta }^{2}}\right)\right)=\\ -\frac{{\left\|Z_{i}\right\|}^{2}}{2\sigma_{\eta }^{2}}\exp\left(-\frac{{\left\|Z_{i}\right\|}^{2}}{2\sigma_{\eta }^{2}}\right)+\exp\left(-\frac{{\left\|Z_{i}\right\|}^{2}}{2\sigma_{\eta }^{2}}\right)\log\left(1+\sum_{\begin{array}{l} j=1\\ i\neq j \end{array}}^{M}\exp\left(-\frac{{\left\|Z_{i}-\tilde{H}\Delta X_{ji}\right\|}^{2}}{2\sigma_{\eta }^{2}}+\frac{{\left\|Z_{i}\right\|}^{2}}{2\sigma_{\eta }^{2}}\right)\right)=\\ -\frac{{\left\|Z_{i}\right\|}^{2}}{2\sigma_{\eta }^{2}}\exp\left(-\frac{{\left\|Z_{i}\right\|}^{2}}{2\sigma_{\eta }^{2}}\right)+\\ \exp\left(-\frac{{\left\|Z_{i}\right\|}^{2}}{2\sigma_{\eta }^{2}}\right)\log\left(1+\sum_{\begin{array}{l} j=1\\ i\neq j \end{array}}^{M}\exp\left(-\frac{{\left\|Z_{j}-\tilde{H}\Delta X_{ji}\right\|}^{2}}{2\sigma_{\eta }^{2}}+\frac{{\left\|Z_{i}\right\|}^{2}}{2\sigma_{\eta }^{2}}\right)\right)>-\frac{{\left\|Z_{i}\right\|}^{2}}{2\sigma_{\eta }^{2}}\exp\left(-\frac{{\left\|Z_{i}\right\|}^{2}}{2\sigma_{\eta }^{2}}\right) \end{array}$$

Considering $\int_{Z_{i}}\frac{1}{{\left(2\pi \sigma_{\eta }^{2}\right)}^{N_{rx}}}\frac{{\left\|Z_{i}\right\|}^{2}}{2\sigma_{\eta }^{2}}\exp\left(-\frac{{\left\|Z_{i}\right\|}^{2}}{2\sigma_{\eta }^{2}}\right)dZ_{i}=N_{rx}$ it is possible to write an upper bound for the capacity of a discrete–continuous MU-MIMO uplink channel:

(18) $$C<\log M=UN_{tx}k_{b}\log2$$

For ergodic capacity calculation, it is necessary to calculate the average value by averaging over the a priori distribution of matrix $\tilde{H}$ elements.

To calculate the average throughput according to formula (17) the Monte Carlo method can be used by averaging simultaneously over random matrix $\tilde{H}$ elements, random Gaussian vectors $Z_{i}∼\mathbb{C}\mathbb{N}\left(0,2\sigma_{\eta }^{2}I_{N_{rx}}\right)$ and random uniformly distributed discrete vectors $X_{i}\in \Omega^{UN_{tx}}$:

(19) $$\begin{array}{l} \bar{C}=\log M-N_{rx}-E_{H,Z_{i},X_{i}}\left\{\log\left(\sum_{j=1}^{M}\exp\left(-\frac{{\left\|Z_{i}-\tilde{H}\left(X_{j}-X_{i}\right)\right\|}^{2}}{2\sigma_{\eta }^{2}}\right)\right)\right\}\approx \\ \log M-N_{rx}-\frac{1}{N_{MK}}\sum_{n=1}^{N_{MK}}\log\left(\sum_{j=1}^{M}\exp\left(-\frac{{\left\|Z^{\left(n\right)}-{\tilde{H}}^{\left(n\right)}\left(X_{j}-X^{\left(n\right)}\right)\right\|}^{2}}{2\sigma_{\eta }^{2}}\right)\right)=\\ -N_{rx}-\frac{1}{N_{MK}}\sum_{n=1}^{N_{MK}}\log\left(\frac{1}{M}\sum_{j=1}^{M}\exp\left(-\frac{{\left\|Z^{\left(n\right)}-{\tilde{H}}^{\left(n\right)}\left(X_{j}-X^{\left(n\right)}\right)\right\|}^{2}}{2\sigma_{\eta }^{2}}\right)\right) \end{array}$$

where ${\tilde{H}}^{\left(n\right)},Z^{\left(n\right)},X^{\left(n\right)}$-*n*-th implementations of random matrices and vectors used for averaging.

The bandwidth of discrete channels is usually measured in bits per second per hertz ((bits/s)/Hz). To obtain the value in these units of measurement, it was necessary to use base 2 logarithm in the initial Equations (4) and (8).

Considering $\log_{2}x=\frac{\log x}{\log2}$ and $M=2^{UN_{tx}k_{b}}$ we can finally write an expression for discrete–continuous MU-MIMO uplink channel capacity and its upper bound:

(20) $$\begin{array}{l} {\bar{C}}_{\left(bps/Hz\right)}=UN_{tx}k_{b}-\frac{N_{rx}}{\log2}-\\ \frac{1}{2^{UN_{tx}k_{b}}}E_{H}\left\{\sum_{i=1}^{2^{UN_{tx}k_{b}}}\int_{Z_{i}}\frac{1}{{\left(2\pi \sigma_{\eta }^{2}\right)}^{N_{rx}}}\exp\left(-\frac{{\left\|Z_{i}\right\|}^{2}}{2\sigma_{\eta }^{2}}\right)\log_{2}\left(\sum_{j=1}^{2^{UN_{tx}k_{b}}}\exp\left(-\frac{{\left\|Z_{i}-\tilde{H}\left(X_{j}-X_{i}\right)\right\|}^{2}}{2\sigma_{\eta }^{2}}\right)\right)dZ_{i}\right\}\approx \\ UN_{tx}k_{b}-\frac{N_{rx}}{\log2}-\frac{1}{N_{MK}}\sum_{n=1}^{N_{MK}}\log_{2}\left(\sum_{j=1}^{2^{UN_{tx}k_{b}}}\exp\left(-\frac{{\left\|Z^{\left(n\right)}-{\tilde{H}}^{\left(n\right)}\left(X_{j}-X^{\left(n\right)}\right)\right\|}^{2}}{2\sigma_{\eta }^{2}}\right)\right)<UN_{tx}k_{b} \end{array}$$

**Figure 2:**
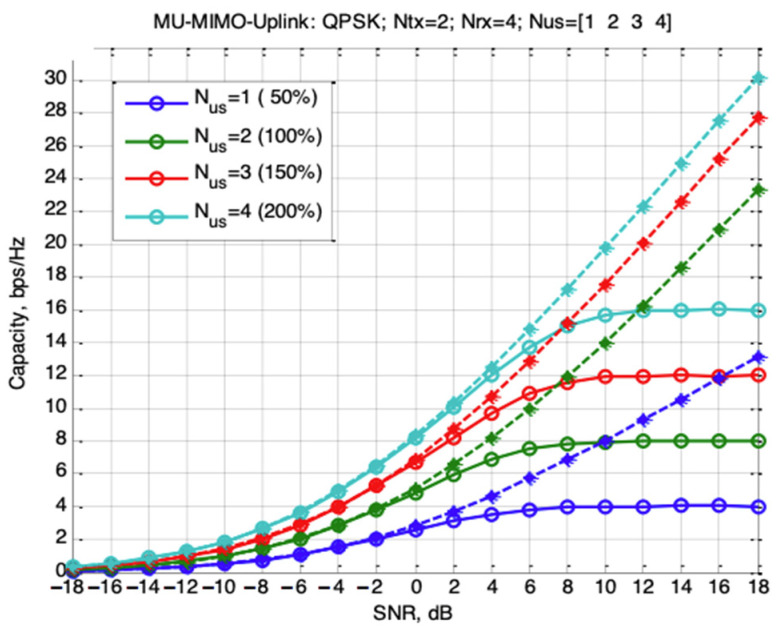
The dependencies of the MU-MIMO uplink channel capacity for discrete–continuous (solid lines) and continuous (dotted lines) channels on the SNR ratio for different numbers of users.

Figure 2 shows the dependencies of capacity of discrete-continuous MU-MIMO uplink channel (solid lines) for the following conditions: $N_{tx}=2,\;\;\;N_{rx}=4$, QPSK modulation, number of users U=[1  2  3  4], which corresponds to the channel load level of 50%, 100%, 150% and 200%. Dotted lines are used to plot the dependencies of the continuous channel bandwidth. Solid and punctured curves of the same color correspond to the same values of number of users.

**Figure 3:**
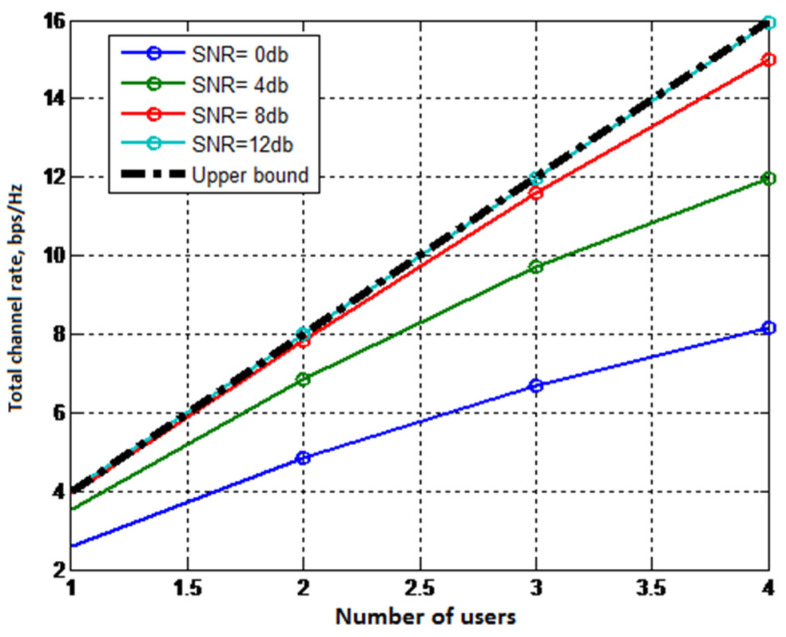
The dependence of MU-MIMO uplink total channel rate for discrete–continuous channel on the number of users of different values of the SNR ratio.

Figure 3 shows the dependencies of the MU-MIMO uplink channel capacity of a discrete–continuous channel on the number of users (system load) at different SNR ratios. The upper border is also shown there (dash dotted black line).

The results on Figure 2 and Figure 3 show that for a discrete–continuous channel with high SNR ratios the total channel rate increases linearly with the number of users. This allows us to conclude that such systems can work in overload mode.

## 4. Simulation of MU-SISO and MU-MIMO Uplink System in Overload Mode

For a demonstration of the potential possibilities of using the overload mode, we consider the case of receiving, on one receiving antenna at the base station, signals from several users, i.e., consider the MU-SISO uplink system $N_{rx}=1,\;\;N_{tx}=1$.

**Figure 4:**
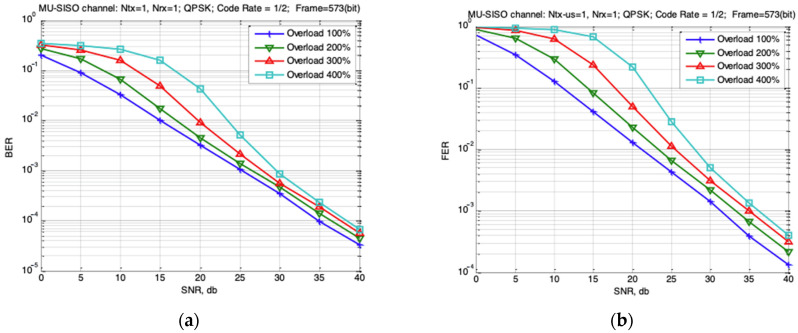
Dependencies of (**a**) BER and (**b**) FER for MU-SISO uplink systems with FEC from SNR for different number of users (different degree of loading).

Figure 4 shows the dependencies of BER and FER for this case with the number of users—1, 2, 3 and 4—which corresponds to the system load of 100%, 200%, 300% and 400%. The follow simulation parameters were used: QPSK modulation and turbo encoder (speed of ½) and Rayleigh fading channel. The optimal maximum likelihood detector was used at the base station receiver side.

The results of Figure 4 show that the MU-SISO uplink system is functional, even under a 400% load. To ensure the level of FER = 0.01 in conditions of load increasing up to 200% and 400%, increasing the SNR ratio up to 2.2 dB and 7 dB respectively is necessary. This is to ensure that the level of FER = 0.001 is necessary to increase SNR ratio up to 2 dB and 5 dB respectively.

**Figure 5:**
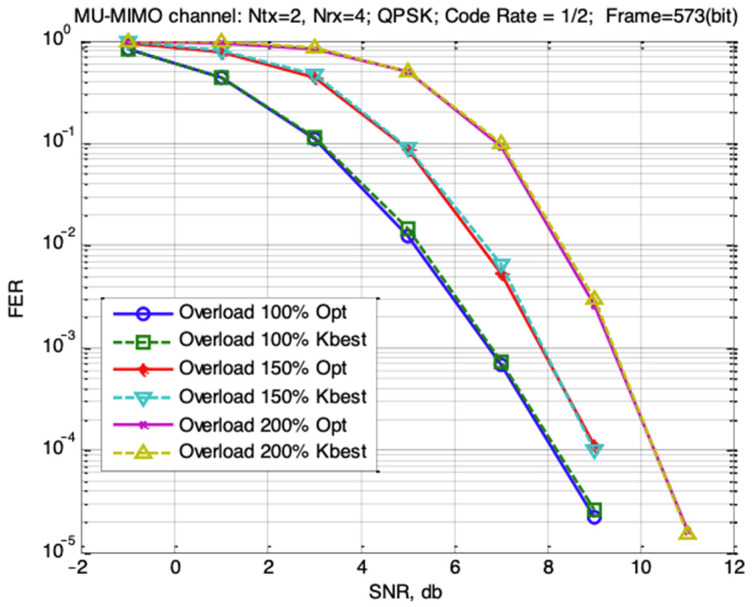
Dependencies of FER from SNR for the MU-MIMO uplink system with different numbers of users using FEC in overload mode.

Figure 5 shows the dependencies of FER from SNR for the MU-MIMO-Uplink system. It is well known that to maximize the efficiency of overloaded systems, it is obligatory to use optimal, or close to optimal, MIMO detectors [12]. For overload systems, the question of computational complexity is the first one. Computational complexity of optimal detector increases proportionally to $2^{UN_{tx}k_{b}}$. Linear detectors (MMSE, ZF) and receivers which use linear detectors (V-BLAST, SIC) [8] in overload conditions become ineffective.

The use of detectors based on QR decomposition (Sphere detector [9] and K-best detector [18]) is not possible, since the decomposition of the channel matrix does not lead to a triangular matrix. However, it exists in a solution based on a K-best algorithm using factorization of MMSE solution [5,18,19,20]. This algorithm was also simulated for the considered MU-MIMO uplink system.

Figure 5 result curves for the optimal MIMO detector are plotted with solid lines, and results from the MMSE-based K-best detector with the parameter $K_{best}=16$ are represented with dotted lines.

From the results shown, it follows that for the considered variations the characteristics of a simpler MMSE-based K-best detector almost match with the characteristics of the optimal ML detector. Increasing the number of users by 1.5 and 2 times (150% and 200% overload) requires an increase in the SNR ration by 1.1 dB and 2.5 dB at the FER = 0.001 level respectively. This confirms the possibility of using such a mode in MU-MIMO uplink systems.

## 5. Conclusions

In the article, based on the analysis of the capacity of a discrete–continuous MU-MIMO uplink channel, the possibility of such systems functioning in overload mode is proved. At the same time, multiuser systems with such a configuration can be considered as communication systems with NOMA technology with separation in power domain, and implemented naturally due to independent Rayleigh fading.

It is shown that at high SNR ratios, the total channel rate of such systems increases proportionally to the total number of transmitting antennas of all users in the system $N_{tx,\Sigma }$, even if it is greater than the number of receiving antennas at the base station $N_{tx,\Sigma }>N_{rx}$, i.e., in overload mode. This conclusion is at odds with the well-established opinion, based on an analysis of the continuous channel capacity, that MIMO systems are inefficient under overload conditions.

Conclusions on the possibility of functioning MU-MIMO uplink systems under overload conditions are confirmed by the simulation results.

## Data Availability

Not applicable.
